# Application of a Method for Measuring the Grindability of Fine-Grained Materials by High-Speed Milling

**DOI:** 10.3390/ma15228085

**Published:** 2022-11-15

**Authors:** Simona Ravaszová, Karel Dvořák, Danute Vaičiukynienė, Martin Sisol

**Affiliations:** 1 Faculty of Civil Engineering, Brno University of Technology, Veveří 331/95, 602 00 Brno, Czech Republic; 2 Faculty of Civil Engineering and Architecture, Kaunas University of Technology, Studentu St. 48, LT513-67 Kaunas, Lithuania; 3 Faculty of Mining, Ecology, Process Control and Geotechnologies, Technical University of Košice, Letna 9, 042 00 Košice, Slovakia

**Keywords:** grindability, grindability index, high-speed milling

## Abstract

This article deals with the development of an alternative method for determining the grindability index of fine-grained materials. This method is inspired by the commercially used VTI method (also known as RTI after the Russian Thermal Energy Institute), which was widely used in Central and Eastern Europe in coal grinding. The disadvantage of the VTI method is that it uses a specific grinding device that otherwise has no other use and nowadays is no longer commonly available. Through the new method, high-energy grinding was performed using a commercially available planetary mill on silicate materials such as limestone, feldspar, corundum, and quartz. The effectiveness of the method was verified on clinker as a representative of widely used materials. The deviation between the grindability index calculated by the origin VTI method and the new developed method was on average approximately 8%; in the case of clinker grinding, it was only 3%. The results showed that the VTI method could be replaced by a new method that uses a modern available planetary mill and laser granulometry to determine the grindability index. The result is a new classification of materials according to their grindability indexes, which is based on the original VTI method.

## 1. Introduction

Crushing processes are widely used in metallurgy, the chemical industry, the building materials industry, the thermal power industry, and many other basic fields of the national economy. These are energy-intensive operations that can account for as much as 50% of the total energy intensity of a given production process [1,2,3,4,5,6]. Therefore, it is important to save energy and reduce energy consumption in the milling process.

Current trends in materials milling include high-energy milling (HEM) methods. The potential applications of HEM cover various industrial fields such as metallurgy [7], the production of various intermetallic and ceramic materials [8], and the preparation of nanomaterials [9]. High-speed grinding (HSG) is a subtype of HEM that is accomplished by delivering large amounts of energy using very short and intense power pulses. The amount of energy that is efficiently transferred to the material is higher with HSG than with conventional milling in mills with identical power inputs. Mechanical activation (MA) has been a well-known technique since prehistoric times, when reactions could be initiated randomly during milling. The first records of systematic investigation of mechanochemistry were recorded by Spring and Lea in the late 19th century [10,11,12]. Nowadays, mechanical activation and mechanical alloying are among the emerging technologies used to produce high value-added particulate products [13,14,15]. Mechanochemical activation is also a widely used method in laboratory syntheses of various materials.

Grindability is one of the important material properties that is monitored in a wide range of industries. It is the ability of a material to break into smaller particles as a result of mechanical work. Grindability is influenced by various factors that can be broadly categorized as material properties, machine parameters, and operating parameters. Material properties such as mineralogy, physical properties, and others cannot be clearly controlled. Milling equipment parameters such as mill type, mill geometry, or mill diameter can only be selected during the set-up of the equipment. Operating parameters such as mill charge, rotation speed, and milling time can be changed and controlled [16,17,18,19,20]. Material that is easy to mill is economically more suitable for the preparation of powder feedstock. Milling is generally analyzed through energy–size relationship models [21].

To determine the amount of energy required for particle milling and the subsequent design of the milling equipment of the plant, it is necessary to find a relationship between the energy consumption and the desired milling result. Several hypotheses are used for this purpose. Unfortunately, even today, the problem of determining the magnitude of the required energy is not satisfactorily solved. Many factors such as the physical and mechanical properties of the material, grain size, and grain shape must be considered in determining the required energy [22,23,24,25,26].

**Figure 1:**
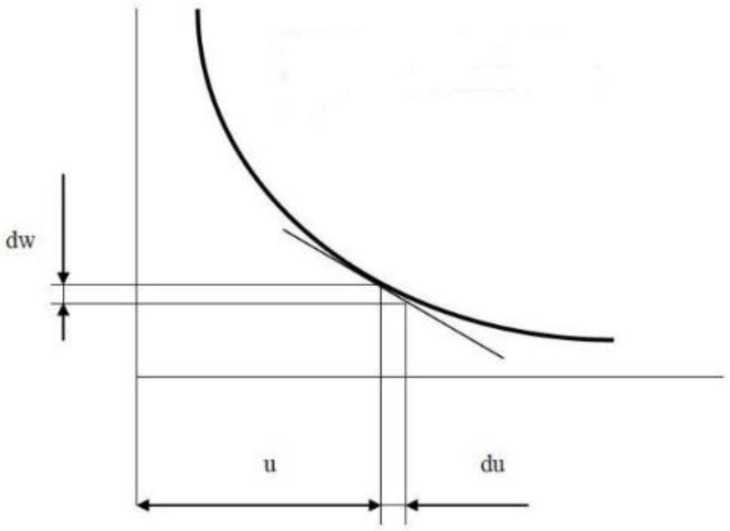
Specific work of uncoupling “w” as a function of particle size “u”.

Kick’s hypothesis of 1885 is based on the premise that the magnitude of the deformation work consumed for uncoupling is the largest item of the total energy consumption. Other energy items are not considered. This means that the deformation work in many cases exceeds the actual theoretical energy consumption to create new surfaces. The hypothesis states that the uncoupling work is directly proportional to the volume of material being uncoupled [27,28,29,30]. Rittinger’s hypothesis of 1867 is based on the idea that the work for uncoupling is different if the newly formed area is the same. The hypothesis is one of the oldest theories and assumes that the work consumed in uncoupling a material is proportional to the increase in the surface area of the material during the process [31]. After conducting practical experiments, it was proved that the hypothesis has no general validity. Comparing the two theories mentioned above, it was found that the surface theory fits the principles of small-scale uncoupling (medium and fine crushing, milling), i.e., in processes in which a large specific surface is formed. In contrast, the bulk theory shows more accurate results for coarse uncoupling. Neither hypothesis fully captures the issue of all physical processes that affect the energy consumption during uncoupling, but they clarify the main principles of the uncoupling mechanism and provide the fundamental energy requirements for uncoupling [32,33]. Bond’s 1952 hypothesis describes the process of diminution from the more modern aspects of the grain uncoupling mechanism. According to this hypothesis, the total amount of energy that is consumed to reduce materials consists of the energy determining the ratio of particle sizes (volume dependent) and the energy required for the newly formed surface. The sum of the total energy required is proportional to the grain volume, but the surface size is also considered. Surface area is accounted for through the necessary stress concentration conditioning the formation of cracks at the grain surface [34,35,36]. Charles’ general equation of disconnection includes all known theories and generalizes them, see Figure 1. It is defined by the variation of the specific uncoupling work as a function of grain size [37,38,39,40]. The amount of work required to uncouple a single grain is shown in the following equations [24,27,30,33]:

(1) $$\mathrm{Kick}\;W_{K}=k_{k}\;.\;U^{3}\;\mathrm{for}\;\mathrm{rough}\;\mathrm{milling},\;$$

(2) $$\mathrm{Bond}\;W_{B}=k_{B}\;.\;U^{2.5}\;\mathrm{for}\;\mathrm{medium}\;\mathrm{rough}\;\mathrm{milling},$$

(3) $$\mathrm{Rittinger}\;W_{R}=k_{R}\;.\;U^{2}\;\mathrm{for}\;\mathrm{high}\;\mathrm{fineness}\;\mathrm{milling},$$

(4) $$\mathrm{In}\;\mathrm{general},\;W=k\;.\;U^{m}\;\mathrm{where}\;m=2\;÷\;3\;$$

Charles’ decoupling equation includes all known equations and theories:

(5) $$d_{w\;}=-C\;.\;\frac{d_{u}}{u^{m}}$$

where *w* is the specific uncoupling work; *u* is the particle size; *C* is a constant of proportionality that characterizes the strength of the material; and *m* is an exponent dependent on the type of energy supplied, e.g., pressure, shear.

Kick’s hypothesis m = 1

(6) $$w=-C\;.\ln\frac{U}{u}=C\;.\ln\frac{u}{U}$$

Rittinger hypothesis, m = 2

(7) $$w=C\;.\;\left(\frac{1}{U}-\frac{1}{u}\right)$$

Bond hypothesis, m = 3/2

(8) $$w=2\;.\;C\;.\;{\left[\frac{1}{\sqrt{u}}\right]}_{u}^{U}=2\;.\;C.\;\left(\frac{1}{\sqrt{U}}-\frac{1}{\sqrt{u}}\right)$$

One of the main technological tests to determine whether a material is suitable for use in technical applications is the grindability test. Milling can be defined as the ability of solids to break down under standardized conditions and can be expressed, for example, as an increase in specific surface area per unit of work expended. The grindability or the index or coefficient of grindability is one of the important material properties that is monitored in a wide range of industries.

The methods for determining the milling indexes are divided into constant fineness methods and constant energy methods [41,42,43]. Constant fineness methods work on the principle of comparing the amount of energy required to disperse the material to a predetermined fineness. The materials being compared have the same input particle size prior to decoupling. These methods are challenging to implement. Constant energy methods work on the principle of comparing the surface increments of the material under test with the surface increment of the standard. It is important to ensure that the same amount of milling energy is supplied for both materials. The energy is determined by the revolutions per minute (rpm) and the grinding time. The surface gain is monitored by sieving the dust onto mesh of the appropriate size.

Of these methods, VTI is the simplest and is the focus of this paper. It is a method widely used in Central and Eastern Europe for the determination of workability in the cement industry. Empirically, this method is based on the above-mentioned hypotheses according to Kick, Rittinger, and Bond. Prior to the actual test, the material shall be treated for initial granulometry in accordance with the requirements of the VTI method, where the required initial grain size is between 1.25 and 3.2 mm. The milling coefficient is denoted as k_VTI_ and according to its value, the material can be classified into different milling grades. All the above milling methods often have one common characteristic, namely, the need for special equipment that has no other use apart from the test in question.

All existing methods dedicated to the grinding process, such as the Hardgrove, Zeisel, Bond, and also VTI methods, are primarily dedicated to coarse grinding. All these methods were primarily designed for industrial grinding of coal in particular. At the same time, their greatest disadvantage is the need for special mills, which of course have no further application. This article aims is to modify the simplest VTI method. The modification is mainly based on the change of the used mill.

The objective is to use the modified method mainly in fine-grained materials. A widely available standardized planetary mill was chosen as the grinding device, which works on the principle of constant work, similar to the porcelain mill of the VTI method. It is a ball mill that mainly grinds by impact, which corresponds to the method of grinding in the VTI method. The goal is to discover whether it is possible to modify the VTI method with a new modified method while maintaining the method of evaluating grindability.

## 2. Materials and Methods

### 2.1. Materials Characterization

For the development of an alternative method for determining the grindability index of fine-grained materials, four materials with different hardness were selected. The suitable minerals were selected based on the Mohs hardness scale, which is based on the relative hardness of 10 basic minerals. Limestone (Mokrá, Devon, 98.98% CaCO_3_) was selected as a representative of the lower hardness materials. Two representatives from the medium hardness area were selected: quartz (Betosan quartz sand, 98% SiO_2_) and feldspar (Dolní Bory site). The representative of hard minerals was technically produced corundum (PKIT Praha s.r.o.). The new developed method was than verified using Portland clinker (Cement Hranice a.s.) as a representative artificial and widely used material. All selected materials were analyzed by X-ray powder diffraction (XRD), X-ray fluorescence (XRF), and hardness according to Vickers (EN ISO 6507-1:2005).

The XRD analysis was performed using a Panalytical Empyrean diffractometer (Malvern Panalytical Ltd., Almelo, the Netherlands). The Θ-Θ reflection Bragg–Brentano para focusing geometry device is equipped with a Cu anode (λ = 1.54184 Å) and programmable divergence slits a PIXcel3D detector with 255 active channels. The settings were 45 kV and 40 mA and the range was 5–80° with a step size of 0.013° and 157 s per step. The total measurement time for each sample was 58 min. The Panalytical HighScore 3 plus software was used to identify the individual phases. The ICSD (released in 2012) was used for a qualitative analysis of the diffraction patterns. Quantification was performed by the Rietveld method with a fundamental parameters approach [44].

The XRF analysis was performed using a Panalytical Axios 2.4 kW sequential wave dispersive spectrometer with Rh anode (Malvern Panalytical Ltd., Almelo, the Netherlands). Samples in the form of borate beads melted in a LeNeo Claisse electric melter at 1065 °C using a mixed flux of Li_2_B_4_O_7_ and LiBO_2_ at a flux/sample ratio of 15:1 for the analysis. The acquired data were evaluated using the SUPERQ V4.0 software. The resulting values represent the average of three independent measurements.

The absolute Vickers hardness measurements of these minerals were conducted in accordance with EN ISO 6507-1:2005: Metallic materials—Vickers hardness test—Part 1: Test method on DuraScan 70 G5. The testing was performed at a test load of 4.903 N, corresponding to a designation of HV 0.5.

### 2.2. Sample Preparation

All the selected raw materials were ground to a very similar initial grain size in between 1.25 and 3.2 mm using the combination of the jaw crusher Retsch BB200 and Retsch RS 200 vibratory disc mill. The separation of the initial grain size was performed using manual sieving. All the samples were next dried in a Binder C170 laboratory dryer at 105 °C for 24 h. The resulting grain size respected the requirements of the VTI method for the initial granulometry.

### 2.3. VTI Method

**Figure 2:**
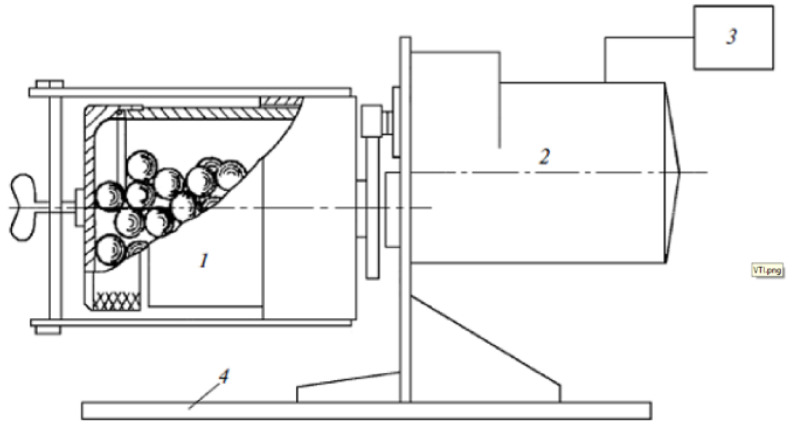
Schematic of a tubular mill, 1—drum, 2—electric engine, 3—control unit, 4—pedestal.

The VTI method was used as a referral method. The test using the VTI method was conducted in a laboratory ball mill GHOST 15489.1-93 with a porcelain drum with a diameter of 270 mm and a length of 210 mm, see Figure 2. The load consisted of 6 kg of grinding porcelain balls with a diameter of 35 mm and 2 kg of porcelain balls with a diameter of 15 mm.

Individual samples were milled for 13.5 min at a speed of 41 rpm. This ensured that the milling conditions of the individual materials were constant. The ground samples were then sieved through a 0.090 mm mesh sieve. The over-sieve share was weighed and converted to a percentage of the original weight. The calculation was performed according to the following formula:

(9) $$k_{\mathrm{VTI}}=2\cdot {\left(\ln\frac{100}{z_{90}}\right)}^{\frac{2}{3}}$$

where:

$$\begin{array}{c} k_{\mathrm{VTI}}\;\mathrm{grindability}\;\mathrm{index},\\ z_{90}\;\mathrm{percentage}\;\mathrm{balance}\;\mathrm{on}\;\mathrm{the}\;\mathrm{sieve}\;(\%). \end{array}$$

Anthracite dust from the Donetsk Basin was used as a standard for this method, with a melting index of k_VTI_ = 1 chosen, representing a 70% residual on a 0.090 mm grid. The evaluation of the test is presented in Table 1.

Based on the value of the grinding index k_VTI_, the individual samples were classified into their respective grinding classes.

For a more detailed investigation into very fine particles, sieve analyses were also performed in the fine fraction area on 0.020 mm, 0.041 mm, and 0.063 mm sieves using an Air Jet matrix sieve vacuum A058-05N (Matest, Arcore, Italy). The over-sieve share fractions were determined for the calculation of the grindability indexes on these sieves.

The granulometry of the selected materials was also determined using a Malvern Mastersizer 2000 laser granulometer with a hydro 2000 G fluid dispersion unit, using 2-isopropanol as the dispersing agent (Malvern Panalytical Ltd., Malvern, Great Britain). The grindability indexes using sieve analysis (k_VTI (SA)_) were compared with the grindability indexes calculated using laser granulometry (k_VTI (LG)_).

### 2.4. THD Method

For the newly developed THD method, the milling of materials in a high-speed laboratory planetary mill (Pulverisette 6, Fritsch, Idar–Oberstein, Germany) was used. The volume of the milling capsule (made of hardened steel with 25 milling steel balls with a diameter of 20 mm) was 500 ml.

The initial granulometry was the same as previously described. The process of the development of this new method was divided into two phases. The first step focused on the design and optimization of the grinding regime. The aim of this phase was to select a regime good enough for comparison with the existing method. A set of measurements was designed in which different milling speeds of 200, 250, 300, 350, and 400 rpm were combined with the constant milling period. The milling time was set at 4 min. This time was selected as a suitable handling period with good compensation for the start-up and run-up of the laboratory mill used. Limestone, feldspar, and quartz were ground at the above combinations of milling regimes. From the laser granulometry tests subsequently conducted, the combination of milling speed and milling time was selected as the most suitable.

The second phase focused on the determination of the grindability index for the new method and its verification. After the evaluation and determination of the optimal milling regime, the suitability of this milling regime was verified on corundum and then on clinker. Subsequently, the milled mineral samples were subjected to sieve analysis (sieve mesh sizes 0.020, 0.041, 0.063, and 0.090 mm) and laser granulometry similar to the VTI method. The grindability index for the THD method k_THD_ was determined according to Equation (9). A comparison of the grindability indexes for the two methods was made and the correlation between the indexes was investigated.

Except for particle size, particle shape was also assessed. For both methods (VTI and THD), images were captured on a Tescan MIRA 3XMU scanning electron microscope (Tescan Brno s.r.o., Brno, Czech Republic).

## 3. Results

### 3.1. Materials Characterization

The following Table 2 shows the chemical analyses of the input material.

This is limestone whose formation dates to the Devonian Period. Based on the XRD analysis, it is a highly pure limestone with a dominant content of calcite CaCO_3_.

Two representatives from the medium hardness area were selected: quartz and feldspar. Their chemical analyses are shown in Table 3 and Table 4.

Based on the XRD analysis, Betosan quartz sand is composed of pure quartz. Feldspar from the Dolní Bory location is high-quality sodium potassium feldspar. Corundum was chosen as the representative of hard minerals. Its chemical analysis is shown in Table 5.

It is technically produced corundum, which is characterized by high purity with an Al_2_O_3_ content of more than 99.7% and a maximum of 0.1% iron. Portland clinker (Cement Hranice a.s.) was selected to verify the grinding regime. The chemical analysis of cement is shown in Table 6.

An industrial clinker is a typical alite Portland clinker with a normal ratio of basic and accessory oxides. The representation of the alite content is 68%.

The values of hardness according to Vickers are summarized in Table 7.

The values show that the materials were chosen appropriately and that their hardnesses determined by this method correspond to the relationship between relative and absolute hardness.

### 3.2. VTI Method

Five sieve analyses were performed for each material and the average over-sieve shares fractions were calculated.

**Figure 3:**
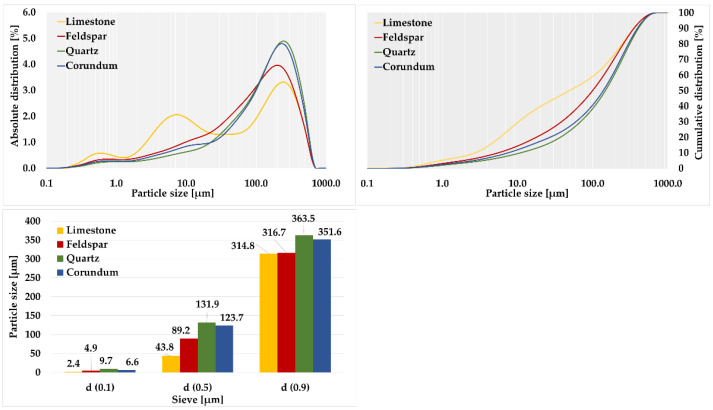
Laser granulometry of quartz, limestone, feldspar, and corundum.

Based on the over-sieve share, see Table 8, the grinding indexes k_VTI_ for each material were determined, see Table 9, as shown in Figure 3.

For corundum, the highest average value of over-sieve share per 0.090 mm mesh was observed, 85.14%. The lowest average value of over-sieve share fraction was observed for limestone, at only 48.80%. The average value of over-sieve share was 61.38% for feldspar and 69.08% for silica.

Based on the calculated grindability indexes and their average values, the individual materials were grouped into appropriate classes. Corundum was the only representative in the category of not easy to grind due to its average value of k_VTI 0.090_ = 0.59. The other materials were categorized as medium grindable materials. Limestone had an average grindability index k_VTI 0.090_ = 1.60, which was close to the threshold for easily grindable materials. For feldspar, an average value of k_VTI 0.090_ = 1.24 was found, while for quartz, an average value of k_VTI 0.090_ = 1.03 was calculated.

Subsequently, laser granulometry was performed to calculate grindability indexes for comparison with the results of sieve analysis, see Table 10 and Table 11.

A high proportion (39.68%) of grains below 0.020 mm was observed in limestone. Quartz had the lowest proportion of 13.89% in this area. For corundum, the content of grains below 0.020 mm was 17.42%, while for feldspar, the value was determined to be 21.29%.

The over-sieve shares fractions that were used to calculate the grindability indexes of each material by laser granulometry were evaluated. For corundum, the highest average over-sieve share per 0.090 mm grid was observed at 86.22%. The lowest observed value of over-sieve share was found for limestone, only 49.72%. The average value of over-sieve share was found to be 62.38% for feldspar and 71.76% for quartz.

According to the calculated grindability indexes, the individual materials were again grouped into the appropriate classes. The classification was identical to the sieve analysis. Corundum was the only representative classified in the category of not easy to grind and the other materials fell into the group of moderately grindable materials.

**Figure 4:**
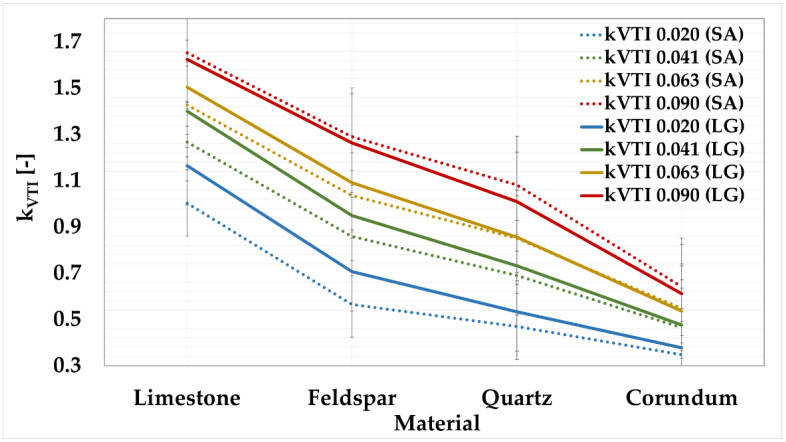
Comparison between k_VTI_ from sieve analysis to k_VTI_ from laser granulometry.

The grindability index values from the sieve analysis were compared with the grindability index values from laser granulometry, as shown in Figure 4.

The above graph shows that, for a given sieve, the lines of the average values of the grindability indexes determined by sieve analysis and laser granulometry are almost identical. For the 0.041 mm, 0.063 mm, and 0.090 mm sieves, this dependence is evident. Table 12 shows the calculated absolute deviations between the grindability indexes from sieve analysis and laser granulometry.

For these sieves, the overall absolute deviations were determined to be the lowest. For the 0.041 mm sieve, the deviation was 7%; for the 0.063 mm sieve, the deviation was 4%; and for the 0.090 mm sieve, the deviation was 5%. For the 0.020 mm sieve, the deviation was set at 18%. When evaluating the deviations relative to the materials, the following values were calculated: feldspar showed the highest variation of 12%; the lowest value was obtained for corundum at 5%; the overall deviation of the k_VTI (LG)_ measurement from the k_VTI (SA)_ was determined to be 8%. This value is satisfactory; thus, it can be concluded that laser granulometry can fully replace the time consuming and inaccurate sieve analysis.

### 3.3. THD Method

**Figure 5:**
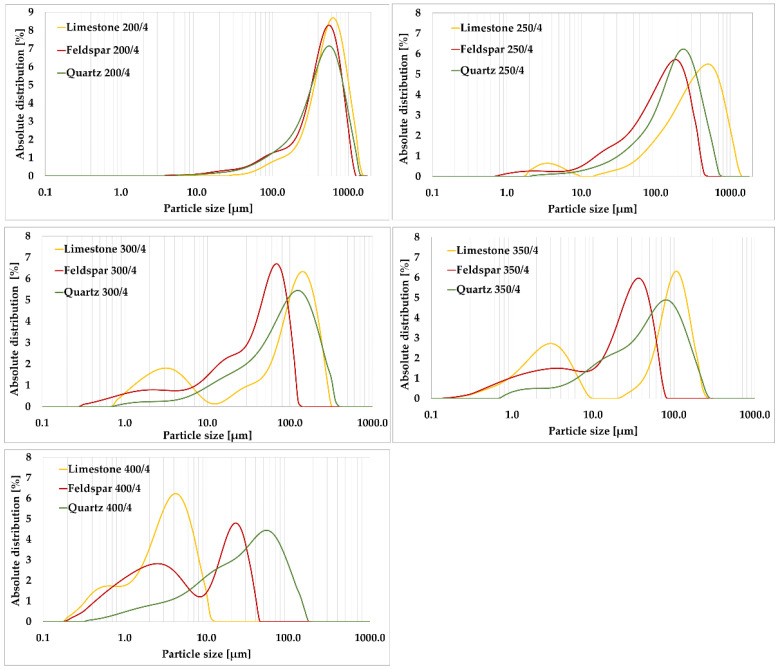
Laser granulometry of limestone, feldspar, and quartz under different milling regimes.

As a first step in the development of the new method, a set of measurements was constructed in which different milling speeds were combined, namely, 200, 250, 300, 350, and 400 rpm. The milling time was set at 4 min. Only limestone, feldspar, and quartz were milled at the above combinations of milling modes, see Figure 5.

From the laser granulometry plot, the curves achieve a steady increase in the content of crushed particles. There is no over-milling of any material. For the 250 rpm milling of limestone, a higher number of ultrafine fractions in the region of 0.001 to 0.010 mm were already produced. Furthermore, the content of ultrafine particles below 0.010 mm was determined to be almost twice as high in limestone as in feldspar. For limestone and feldspar, the content of particles below 0.020 mm was 6.12% and 8.98%, respectively. The difference was in the representation of individual particle sizes below 0.020 mm. For quartz, the value was determined to be 3.76%. Feldspar showed the highest value of 8.94% for grain size 0.063 to 0.090 mm. The lowest value was determined for limestone at 3.09%. It was verified that the milling time was selected optimally as there was no mineral over-milling and agglomerate formation.

At a milling speed of 300 rpm, at first glance, the graph shows a uniform grain size reduction in feldspar and quartz. For limestone, greater amounts of ultrafine fractions in the region of 0.001 to 0.010 mm were already formed. Furthermore, the content of ultrafine particles below 0.010 mm was almost twice as high in limestone as in feldspar. For limestone and feldspar, the content of particles below 0.020 mm was 27.10% and 28.85%, respectively. These values were almost identical. The difference was in the representation of individual particle sizes below 0.020 mm. For quartz, the value was determined to be 12.99%. At 13.28%, feldspar showed the highest value of grain size content of 0.063 to 0.090 mm. The lowest value was determined for limestone at 5.32%, where there was already a large refinement of particles.

At 350 rpm, the graph showed the formation of agglomerates in limestone, which is undesirable (an area outside the validity of Rittinger’s hypothesis). In feldspar and quartz, agglomerates do not form. Furthermore, the content of ultrafine particles below 0.020 mm was found to be almost twice as high in limestone and feldspar. This value was 42.08% for limestone and 43.66% for feldspar. For quartz, this value was determined to be 23.60%. Quartz had the highest value of grain size at 0.063 to 0.090 mm, at 13.85%. The lowest value was determined for feldspar at 6.99%. Furthermore, it was evident from the graph that the curves could not be correctly compared with each other as they were very heterogeneous. A single quartz showed a uniform increase in the content of crushed particles. For both limestone and feldspar, the reworking to form ultrafine fractions was already evident. Almost the same trend was then observed at a higher grinding speed of 400 rpm.

**Figure 6:**
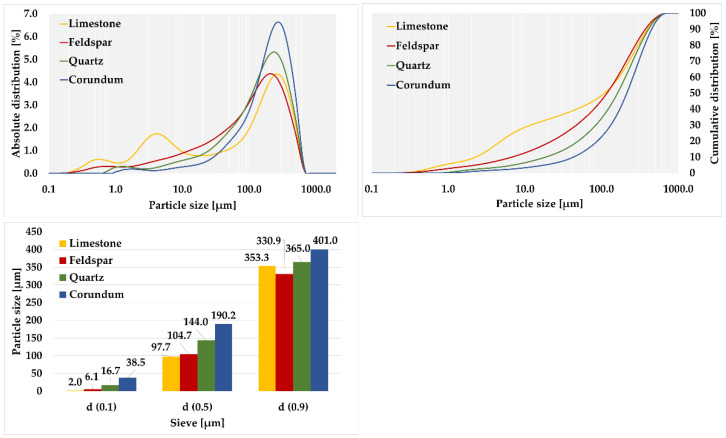
Laser granulometry of materials in milling mode at 250 rpm.

To determine the optimum milling mode, 250 rpm was selected and the suitability of this was verified on corundum. A set of five measurements was taken for each calibration material, averaged, and plotted on a graph, see Figure 6.

Subsequently, the over-sieve share percentages were determined, see Table 13, from which the grindability indexes k_THD_ were determined.

The Figure 6 shows that the individual materials were milled uniformly without the formation of agglomerates, i.e., within the validity of Rittinger’s hypothesis.

For corundum, the highest average value of over-sieve shares was observed at 0.090 mm, with a value of 80.44%. The lowest observed mean value of over-sieve shares fraction was observed for limestone, with the value being only 52.71%. The average value of over-sieve shares was found to be 57.27% for feldspar and 68.97% for quartz. When comparing the shares, it was evident that the values of the over-sieve shares for the pure minerals corresponded to the values of relative hardness. It was evaluated that the average values of over-sieve shares for 0.020 mm, 0.041 mm, and 0.063 sieves showed the same trend as for the 0.090 mm sieve. The highest over-sieve values are for corundum and the lowest are for limestone.

From the graph, the following average values of the grindability indexes were found, see Table 14. The highest values were again achieved by corundum with an average value of k_THD 0.090_ = 0.72, which was close to the limiting region for medium-to-non-easily grindable materials in the VTI method. Limestone had the lowest average grindability index value of k_THD 0.090_ = 1.49, which was close to the limit for easily grindable materials in the VTI method. For feldspar, an average value of k_THD 0.090_ = 1.35 was found, while for quartz, an average value of k_THD 0.090_ = 1.03 was calculated.

Another objective in the development of the new method was to look for a possible dependence between the values obtained by the VTI method and the values obtained by the developed THD method. The trend lines for the grindability indexes for the different materials, which were determined by laser granulometry for both methods, were monitored. The values of the basic calibration minerals were used for comparison.

Table 15 shows the absolute values of the measurement deviations. The k_THD (LG)_ indexes show a higher precision of measurement than the k_VTI (LG)_ indexes.

The graph and table show that the deviations are not very large. The total deviations in absolute value have been determined. For the 0.041 mm sieve, the deviation was only 5%; for the 0.020 mm sieve, the deviation was 6%; for the 0.063 mm sieve, the deviation was 9%, with the largest deviation recorded for the 0.090 mm sieve at 11%. When evaluating the deviations relative to the materials, the following values were calculated. The lowest deviations were obtained for limestone and quartz, namely, 4%. The highest value was obtained for feldspar, at 14%. The overall deviation of the k_VTI (LG)_ measurement from k_THD (LG)_ was determined to be 8%. This value is satisfactory; thus, it can be concluded that the laser granulometries correspond with each other.

**Figure 7:**
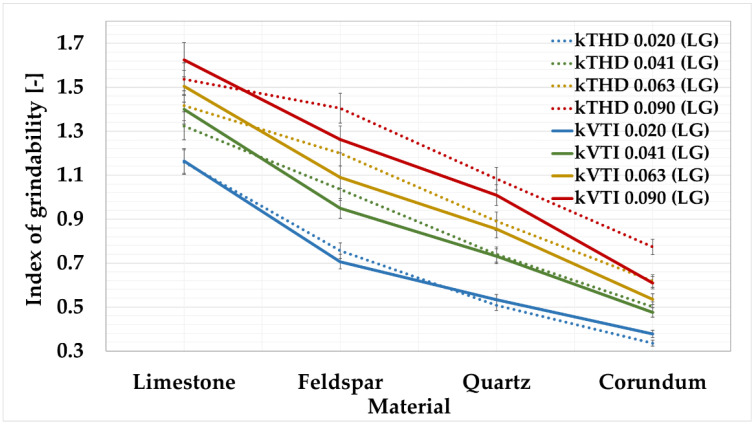
Comparison of grindability indexes by laser granulometry for VTI and THD methods.

It will be possible to maintain the limits of the grindability grades for the 0.090 mm sieve, which are processed for the VTI method. For other sieves, the following recalculation must be made, as shown in Table 16. These limits have been adjusted according to Table 14 and Figure 7 to be consistent with the classification of minerals from the original VTI method.

**Figure 8:**
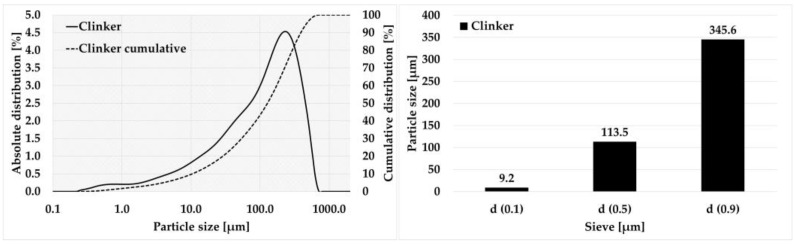
Laser granulometry of clinker (THD method).

The performance of the newly developed THD method was also verified on Portland clinker. The results of the laser analysis are shown in the Figure 8.

For clinker, the average value of the over-sieve share on the 0.090 mm grid was found to be 60.49%, see Table 17. The assumption that the value would be between the values for feldspar and quartz was confirmed. Most likely, this phenomenon was caused by finer milling of clinker, when the grains of the minerals themselves, which are compact, could already be milled. The average value of the grindability index was determined to be k_THD 0.090_ = 1.26. Here again, the assumption was that the value would be between that of feldspar and quartz, which was again confirmed.

The deviation between the grindability index calculated from the VTI method and the THD method was only 3%, see Table 18.

Based on the new classification, as shown in Table 16, clinker is classified into the group of moderately grindable materials as in the case of the VTI method. It can be concluded that the given grinding conditions for the THD method also work for clinker milling.

As additional information, particle morphology was performed by SEM. Microscopic images of the individual materials ground by both methods are shown in the following figures at 1000× magnification.

**Figure 9:**
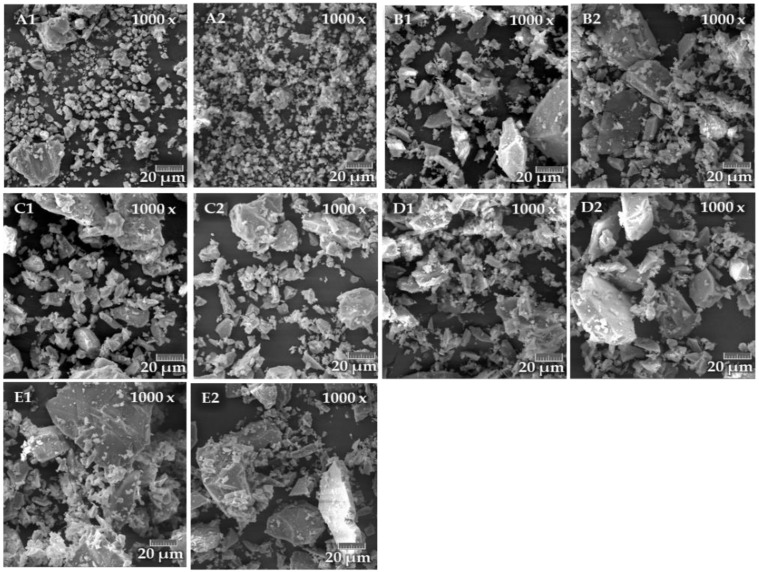
Comparison of the materials shape from the VTI and THD methods. (**A1**): limestone VTI, (**A2**): limestone THD; (**B1**): feldspar VTI, (**B2**): feldspar THD; (**C1**): clinker VTI, (**C2**): clinker THD; (**D1**): quartz VTI, (**D2**): quartz THD; (**E1**): corundum VTI, (**E2**): corundum THD.

Electron microscope images, see Figure 9, show a larger volume represented by smaller particles in the newly developed THD method. In the traditional VTI method, coarser particles are represented. This is most evident in limestone.

Furthermore, the grains of corundum and quartz are pointed, which indicates their hardness and brittleness. In clinker, the rounded edges of the grains are noticeable compared to quartz or corundum. In feldspar, the grains are pointed. They have an elongated shape due to feldspar’s preferential cleavage. In limestone, the grains are rounded because it is a soft mineral.

## 4. Discussion

Firstly, the results were evaluated according to the standard procedure of the VTI method using sieve analysis. Comparison of the average values of over-sieved shares on 0.090 mm mesh (sieve analysis) showed an increasing trend. The hardest mineral (corundum) had the highest over-sieve share and the softest material (limestone) the lowest (values correspond to the relative and absolute hardness of the minerals). Subsequently, it was evaluated that the average values of the over-sieve shares for the 0.020 mm, 0.041 mm, and 0.063 sieves show the same trend as for the 0.090 mm sieve. This is because they are pure and compact minerals. Corundum has been classified as the only representative in the category of materials that are not easy to grind. The other materials were categorized as medium grinding materials. The hardest mineral (corundum) had the lowest grindability index value and the softest material (limestone) had the highest (the values correspond to the relative and absolute hardness of the minerals).

Secondly, laser granulometry evaluation was performed. Similar trends to the sieve analysis were confirmed. Only for the 0.041 mm sieve size were the average over-sieve value, and subsequently the grinding index value, in the range of values after feldspar and quartz. The reason considered is based on the same reasons as for the evaluation of the sieve analysis. Another factor is that the evaluation by laser granulometry will be more accurate.

The next step in the VTI method was to compare the results obtained by sieve analysis and laser granulometry. When comparing the values, it was generally observed that for the sieves in question, the lines of the average grindability index values obtained by sieve analysis and laser granulometry were almost identical. This dependence is most obvious for the 0.041 mm, 0.063 mm, and 0.090 mm sieves. For these sieves, the total deviations in absolute value were determined to be the lowest. The overall deviation of the k_VTI (LG)_ measurement from the k_VTI (SA)_ was determined to be 8%, indicating that laser granulometry can fully replace sieve analysis. Furthermore, it can be stated that high deviations may be due to inaccurate measurements by sieve analysis. Another possible source of error is the applicability of the method. The VTI method is designed for milling soft and medium–hard materials. Corundum is a hard material, and it is possible that the milling balls have been soaked with corundum.

For the new THD method developed, a milling mode of 250 rpm for 4 min was selected as the optimal mode. The lower speed allows more efficient use of milling energy, and the longer milling time eliminates the effect of mill ramp-up and braking.

The next step in the developed method was to evaluate the grindability indexes. For corundum, the highest average value of over-sieve shares was observed on all screens. The lowest observed average over-sieve value was found for limestone. Furthermore, the average over-sieve share values for 0.020 mm, 0.041 mm, and 0.063 sieves show the same trend as that of the 0.090 mm sieve.

For the grindability indexes, the highest average values were again achieved by corundum with an average value of k_THD 0.090_ = 0.72, which was close to the limiting region for moderately to poorly grinding materials in the VTI method. Limestone had the lowest average grindability index value of k_THD 0.090_ = 1.49, which was close to the limit for easily grindable materials in the VTI method.

Therefore, it can be concluded that it will be possible to maintain the 0.090 mm sieve limit for the new method.

In the last part, the grindability indexes determined by the commercial VTI method were compared with the results obtained by the developed THD method. The continua of the values were similar. The k_THD (LG)_ indexes showed a higher accuracy of measurement than the k_VTI (LG)_. This may be due to particles being released into the samples during the sieve analysis; despite all efforts, the sieves were not cleaned perfectly.

It was found that the deviations are not very large. For the 0.041 mm and 0.020 mm sieves, the deviations were very similar at 5–6%. The overall deviation of the k_VTI (LG)_ from the k_THD (LG)_ index measurement was determined to be 8%. This value is satisfactory. The high deviation values for corundum are most likely due to the following reasons.

Primarily, the VTI method is designed for milling soft and medium–hard materials. Corundum is a hard material, and it is possible that the porcelain grinding balls and capsule have been soaked with corundum, whereas the capsules and grinding balls used for the development of the new laboratory planetary mill method are made of durable steel.

From the above findings, it can be concluded that it will be possible to maintain the limits of the grindability grades for the 0.090 mm sieve that are processed for the VTI method. For the other sieves, a recalculation had to be made.

The efficiency of the THD method was verified by clinker grinding. Based on the comparison of the grindability index between the VTI and THD methods and the absolute deviations between the indexes, which amounted to only 3%, it can be concluded that the THD method is also suitable for cement grinding.

Furthermore, the expected grain shape was verified by microscopic images. The grains were sharp-angled for hard materials and rounded for soft materials.

## 5. Conclusions

The aim of this article was to discover whether it is possible to modify the well-known VTI method, which is used for testing the grindability of materials. The modification of the method consisted in changing the grinding device and the method of the evaluation mechanism. The originally used porcelain mill would be replaced by an available planetary laboratory mill, while the time-consuming sieve analysis to determine the grindability index would be replaced by a faster laser granulometry. It was found that the grindability index trends from both methods are very similar. From this, it can be concluded that these two methods can be used interchangeably. The functionality of the new method was also verified on clinker, where the difference in grindability between the VTI method and the new THD method is only 3%.

The result is a method for fine-grain grinding that uses a modern laboratory mill that is widely available in laboratory practice, with precise laser granulometry used to determine the grindability index.

## Figures and Tables

**Table 1:** Grindability class based on the grindability index value.

Index k_VTI_	>1.8	0.8–1.8	<0.8
Grindability	easily grindable	moderately grindable	difficultly grindable

**Table 2:** Chemical composition of limestone.

Components	CaO	SiO_2_	Al_2_O_3_	Fe_2_O_3_	MgO	TiO_2_	K_2_O	Na_2_O	P_2_O_5_	Other
Content [%]	71.7	3.1	1.7	0.4	0.8	0.1	0.1	0.1	0.5	0.2

**Table 3:** Chemical composition of quartz.

Components	CaO	SiO_2_	Al_2_O_3_	Fe_2_O_3_	MgO	TiO_2_	K_2_O	Na_2_O	P_2_O_5_
Content [%]	0.2	93.6	4.7	0.5	0.1	0.1	2.1	0.6	0.03

**Table 4:** Chemical composition of feldspar.

Components	CaO	SiO_2_	Al_2_O_3_	Fe_2_O_3_	MgO	TiO_2_	K_2_O	Na_2_O	P_2_O_5_	Other
Content [%]	0.8	62.6	18.9	0.5	0.1	0.1	10.7	3.3	0.6	0.1

**Table 5:** Chemical composition of corundum.

Components	CaO	SiO_2_	Al_2_O_3_	Fe_2_O_3_	MgO	TiO_2_	K_2_O	Na_2_O	P_2_O_5_
Content [%]	0.1	0.5	97.1	0.02	0.1	0.1	0.1	0.1	0.02

**Table 6:** Chemical composition of clinker.

Components	CaO	SiO_2_	Al_2_O_3_	Fe_2_O_3_	MgO	SO_3_	K_2_O	Na_2_O	Other
Content [%]	57.9	17.6	4.5	3.5	1.3	3.3	0.9	0.0	0.5

**Table 7:** Vickers hardness of materials.

Material	Diagonal	Method	Designation	Hardness
Limestone	85.5	HV 0.5	127 HV 0.5	127
Feldspar	36.7		731 HV 0.5	731
Quartz	28.3		1160 HV 0.5	1160
Corundum	21.4		2024 HV 0.5	2024

**Table 8:** Sieve analysis of materials after grinding, over sieve (%).

	0.020	0.041	0.063	0.090
Limestone	71.98	62.21	56.53	48.80
Feldspar	87.76	77.34	70.69	61.38
Quartz	90.87	83.47	77.31	69.08
Corundum	94.40	90.97	88.69	85.14

**Table 9:** Calculated k_VTI (SA)_ (-) indexes according to Equation (9).

	0.020	0.041	0.063	0.090
Limestone	0.95	1.22	1.38	1.60
Feldspar	0.51	0.81	0.99	1.24
Quartz	0.42	0.64	0.80	1.03
Corundum	0.30	0.41	0.50	0.59

**Table 10:** Laser granulometry analysis of materials after grinding, over sieve (%).

	0.020	0.041	0.063	0.090
Limestone	65.96	57.45	53.80	49.72
Feldspar	82.85	73.97	68.70	62.38
Quartz	88.82	81.99	77.47	71.76
Corundum	93.58	90.65	88.72	86.22

**Table 11:** Calculated k_VTI (LG)_ (-) indexes according to Equation (9).

	0.020	0.041	0.063	0.090
Limestone	1.11	1.35	1.45	1.57
Feldspar	0.66	0.90	1.04	1.21
Quartz	0.48	0.68	0.80	0.96
Corundum	0.33	0.43	0.49	0.56

**Table 12:** Absolute deviation between k_VTI (SA)_ and k_VTI (LG)_ (%).

	0.020 mm	0.041 mm	0.063 mm	0.090 mm	Deviation
Limestone	17	11	6	2	9
Feldspar	28	11	6	2	12
Quartz	15	7	1	7	7
Corundum	10	3	2	5	5
Deviation	18	7	4	5	8

**Table 13:** Laser granulometry analysis of materials after grinding, over sieve (%).

	0.020	0.041	0.063	0.090
Limestone	66.14	60.14	56.86	52.71
Feldspar	81.06	70.77	64.67	57.27
Quartz	89.63	81.68	76.15	68.97
Corundum	94.75	89.86	85.94	80.44

**Table 14:** Calculated k_THD_ indexes (%) according to Equation (9).

	0.020	0.041	0.063	0.090
Limestone	1.11	1.27	1.37	1.49
Feldspar	0.71	0.99	1.15	1.35
Quartz	0.46	0.69	0.84	1.03
Corundum	0.29	0.45	0.57	0.72

**Table 15:** Absolute deviation between k_VTI (LG)_ and k_THD (LG)_ (%).

	0.020 mm	0.041 mm	0.063 mm	0.090 mm	Deviation
Limestone	0	6	6	5	4
Feldspar	7	9	10	10	9
Quartz	4	1	5	7	4
Corundum	14	4	14	22	14
Deviation	6	5	9	11	8

**Table 16:** New classification of material based on grindability index according to the new THD method.

Index k_THD_	Difficultly Grindable	Moderately Grindable	Easily Grindable
Up to 0.090 mm	<0.80	0.80–1.80	>1.80
Up to 0.063 mm	<0.65	0.65–1.65	>1.65
Up to 0.041 mm	<0.50	0.50–1.50	>1.50
Up to 0.020 mm	<0.35	0.35–1.35	>1.35

**Table 17:** Laser granulometry analysis of clinker, over sieve (%).

	0.020	0.041	0.063	0.090
Clinker	84.62	73.93	67.70	60.49

**Table 18:** Comparison of grindability indexes k_THD_ and k_VTI_ (-) and classification of clinker.

Clinker	**k_THD 0.020_**	**k_THD 0.041_**	**k_THD 0.063_**	**k_THD 0.090_**	moderately grindable
	0.61	0.9	1.07	1.26	
	**k_VTI 0.020_**	**k_VTI 0.041_**	**k_VTI 0.063_**	**k_VTI 0.090_**	moderately grindable
	0.59	0.87	1.04	1.23	
Deviation	3	3	3	3	

## Data Availability

The data presented in this paper are available upon request from the corresponding author.
